# Effect of long‐term organic and mineral fertilization strategies on rhizosphere microbiota assemblage and performance of lettuce

**DOI:** 10.1111/1462-2920.14631

**Published:** 2019-04-29

**Authors:** Soumitra Paul Chowdhury, Doreen Babin, Martin Sandmann, Samuel Jacquiod, Loreen Sommermann, Søren Johannes Sørensen, Andreas Fliessbach, Paul Mäder, Joerg Geistlinger, Kornelia Smalla, Michael Rothballer, Rita Grosch

**Affiliations:** ^1^ Helmholtz Zentrum München, German Research Center for Environmental Health Institute of Network Biology Germany; ^2^ Federal Research Centre for Cultivated Plants, Julius Kühn‐Institut Institute for Epidemiology and Pathogen Diagnostics Braunschweig Germany; ^3^ Plant‐Microbe Systems Leibniz Institute of Vegetable and Ornamental Crops Großbeeren Germany; ^4^ Agroécologie, AgroSup Dijon, INRA University Bourgogne Franche‐Comté France; ^5^ Institute of Bioanalytical Sciences Anhalt University of Applied Sciences Bernburg Germany; ^6^ Department of Biology, Section of Microbiology University of Copenhagen Copenhagen Denmark; ^7^ Department of Soil Sciences Research Institute of Organic Agriculture (FiBL) Frick Switzerland

## Abstract

Long‐term agricultural fertilization strategies gradually change soil properties including the associated microbial communities. Cultivated crops recruit beneficial microbes from the surrounding soil environment *via* root exudates. In this study, we aimed to investigate the effects of long‐term fertilization strategies across field sites on the rhizosphere prokaryotic (*Bacteria* and *Archaea*) community composition and plant performance. We conducted growth chamber experiments with lettuce (*Lactuca sativa* L.) cultivated in soils from two long‐term field experiments, each of which compared organic versus mineral fertilization strategies. 16S rRNA gene amplicon sequencing revealed the assemblage of a rhizosphere core microbiota shared in all lettuce plants across soils, going beyond differences in community composition depending on field site and fertilization strategies. The enhanced expression of several plant genes with roles in oxidative and biotic stress signalling pathways in lettuce grown in soils with organic indicates an induced physiological status in plants. Lettuce plants grown in soils with different fertilization histories were visibly free of stress symptoms and achieved comparable biomass. This suggests a positive aboveground plant response to belowground plant–microbe interactions in the rhizosphere. Besides effects of fertilization strategy and field site, our results demonstrate the crucial role of the plant in driving rhizosphere microbiota assemblage.

## Introduction

Long‐term soil fertilization strategies play a significant role in the alteration of soil biological properties, and thus may affect soil functioning and quality (Bünemann *et al*., 2018). Several studies showed that soil microbial communities are influenced by long‐term fertilization strategies (Hartmann *et al*., 2015; Francioli *et al*., 2016; Lupatini *et al*., 2017). The use of mineral fertilizers in conventional agriculture has contributed to increased crop productivity over the last decades (Robertson and Vitousek, 2009). However, the long‐term use of mineral fertilizers is associated with changes in soil pH (Geisseler and Scow, 2014), which has important implications on, e.g. bacterial community compositions (Delgado‐Baquerizo *et al*., 2018). While mineral fertilizers are simple molecules that are directly available for plants, organic fertilizers, containing nutrients derived from plant or animal sources (e.g. farmyard manure, compost, digestates or sewage sludge), consist of complex molecules, like humic substances and lignocellulose. These compounds do serve not only the plants but also the soil microbiota as nutrient source and increase the soil organic matter content (Mäder *et al*., 2002; Liang *et al*., 2012). Accordingly, it has been reported that soils under long‐term organic fertilization strategies harbour higher microbial biomass (Fließbach *et al*., 2007), higher microbial species richness (Esperschütz *et al*., 2007; Hartmann *et al*., 2015; Schmid *et al*., 2017), enhanced microbial activity (Lori *et al*., 2017) and an increased abundance of plant beneficial microbes (Francioli *et al*., 2016). However, the effect of agricultural practices on the soil microbiota is not consistent across published reports and greatly depends on the pedoclimatic context and the overall agricultural strategy applied at the site.

Besides the soil microbial seed bank, the plant genotype also determines to a large extent the assemblage of the rhizosphere microbiota. A subset of the bulk soil (BS) microbes can proliferate in the vicinity of the root in response to plant‐derived rhizodeposits and some of them can overcome the plant defence system to colonize the inside of plants. This process is discussed as recruitment of beneficial microorganisms by the plant (Berg and Smalla 2009; Philippot *et al*., 2013). The root‐associated microbiota is assumed to be crucial for plant health (Berg *et al*., 2015). In recent years, it was shown that beneficial rhizosphere microorganisms can improve the plant health via induced systemic resistance (ISR) – an important mechanism by which the whole plant is primed for enhanced defence against various plant pathogens (reviewed by Pieterse *et al*., 2014, Pérez‐Jaramillo *et al*. 2016). Interactions between plant roots and beneficial rhizosphere microbes can result furthermore in altered growth, functional traits and nutritional quality of aboveground plant parts (Lau and Lennon 2011; Panke‐Buisse *et al*., 2015). The effects of agricultural management practices such as long‐term mineral or organic fertilization are reflected in the form of specific soil microbial communities or nutrient availability, and are therefore expected to affect belowground plant–microbe interactions and potentially aboveground plant performance (van der Putten *et al*., 2016).

Prokaryotes (*Bacteria* and *Archaea*) contribute to a large part to the enormous biodiversity observed in soils and they are involved in various important ecosystem functions such as nutrient cycling and plant health (Bardgett and van der Putten, 2014). In this study, we investigated the rhizosphere prokaryotic community assemblage and aboveground performance of lettuce cultivated in soils conditioned by different fertilization strategies in the long term. We hypothesized that long‐term fertilization shapes the soil prokaryotic communities and the rhizosphere microbiota assemblages. Furthermore, we assumed that lettuce growth and performance will differ depending on the soil fertilization history. Experiments were carried out with two soil types from LTEs geographically located in different areas, each with long‐term organic and mineral fertilization history. We conducted an exploratory research using a multidisciplinary approach with lettuce cultivated in growth chambers under controlled conditions to avoid influences of interfering parameters (priority effects, microbial successions, environmental fluctuations). High‐throughput sequencing of 16S rRNA gene amplicons was used to characterize BS and rhizosphere prokaryotic community compositions as well as the influence of the field site and fertilization strategies. Moreover, we used this model system to study the aboveground plant growth and health in terms of changes in expression of several lettuce genes with roles in oxidative and biotic stress signalling pathways in the shoots. Our results showed that long‐term fertilization and field site legacies shaped the soil and rhizosphere prokaryotic communities; however, the selective pressure of the plant drove the rhizosphere microbiota assemblage and may have facilitated the observed proper plant development.

## Results

### *Soil physicochemical analysis, lettuce growth and nutrient content*

Soils were sampled at two LTE sites located in Thyrow, North German Plain (52°16’N, 13°12′E; designated as HUB‐LTE, established in 2006) and in subalpine Therwil, Switzerland (47°30’N, 7°33′E; designated as DOK‐LTE, established in 1978). In both LTEs, soils were collected from two different long‐term fertilization strategies [mineral (CONMIN, HU‐min) versus organic (BIODYN2, HU‐org)] and used for growth chamber experiments (Table S1). Analysis of physicochemical parameters in BSs revealed a fertilization legacy (Table 1). More fertilization‐dependent differences among physicochemical soil parameters were found in DOK‐LTE (pH, C_total_, C_org_N_total_, NO_3_^−^—N, P, K, Fe, Mn, Zn) compared with HUB‐LTE (Table 1). Zinc concentration was significantly higher in both organic soils than mineral.

**Table 1:** Analysis of physicochemical parameters in BSs after incubation time of 10 weeks in the growth chamber.

Site	Treatment	pH	C total [mg/100‐g soil]	Corg [%]	N total [mg/100‐g soil]	NO_3_^−^—N [mg/100‐g soil]	C/N	P [mg/100‐g soil]	K [mg/100‐g soil]	Mg [mg/100‐g soil]	Na [mg/100 g soil]	Cu [mg/100 g soil]	Fe [mg/100 g soil]	Mn [mg/100 g soil]	Zn [mg/100 g soil]	Salt [mg/100 g soil]	EC [μS/cm]
DOK	CONMIN	5.64 ± 0.04b	1193.5 ± 5.27b	1.19 ± 0.01b	200.36 ± 11b	101.95 ± 6.32b	6.01 ± 0.3a	2.54 ± 0.02b	6.46 ± 0.29b	25.45 ± 3.51a	6.65 ± 0.9a	0.55 ± 0.02a	16.48 ± 0.18a	23.28 ± 0.46b	0.42 ± 0.02b	202.36 ± 35.56a	765.59 ± 129.91a
	BIODYN2	6.61 ± 0.1a	1670.12 ± 7.18a	1.67 ± 0.01a	256.25 ± 15.07a	207.52 ± 32.83a	6.59 ± 0.42a	2.85 ± 0.06a	9.01 ± 0.53a	19.91 ± 2.08a	8.51 ± 0.78a	0.53 ± 0a	14.58 ± 0.17b	28.73 ± 1.03a	0.78 ± 0.01a	242.86 ± 43.25a	909.67 ± 157.99a
HUB	HU‐min	6.42 ± 0.15a	736.45 ± 5.71a	0.76 ± 0.01a	66.24 ± 1.93a	24.86 ± 9.48a	11.13 ± 0.24a	9.5 ± 0.27a	11.77 ± 0.37a	4.02 ± 0.15a	2.75 ± 0.25a	0.13 ± 0b	14.9 ± 0.12a	4.36 ± 0.06a	0.33 ± 0b	50.3 ± 8.68a	207.19 ± 31.5a
	HU‐org	6.42 ± 0.06a	712.18 ± 6.98a	0.74 ± 0a	73.54 ± 7.12a	53.55 ± 17.89a	9.93 ± 0.85a	10.17 ± 0.17a	6.76 ± 1.23b	4.35 ± 0.94a	2.93 ± 0.4a	0.19 ± 0.01a	15.28 ± 0.17a	4.63 ± 0.11a	0.38 ± 0.01a	67.68 ± 20.8a	271.44 ± 75.75a

The means (*n* = 4, except HU‐min: *n* = 3) per treatment are shown along with standard errors. Different lowercase letters indicate significant differences between organic versus mineral fertilization tested separately for each long‐term experimental site by pairwise *t*‐test comparisons of least squares means.

Lettuce plants were harvested after 10 weeks at BBCH19 growth stage. According to analysis of variance (ANOVA), plants grown in soils from HUB‐LTE showed a significantly (p < 0.05) higher shoot fresh mass than plants grown in soils from DOK‐LTE (Table 2). Regarding the root fresh mass, there were no significant differences between field sites but the same tendency appeared as for the shoots. In contrast to DOK‐LTE soils, a significantly higher shoot and root fresh mass was observed in mineral versus organic fertilized soils from HUB‐LTE. Corresponding to fertilization‐dependent differences among soil parameters, more differences were observed in the plant shoot nutrient content in DOK‐LTE than in HUB‐LTE (Table 2). Leaf nitrogen content was lower and C/N ratio higher in lettuce grown in BIODYN2 than CONMIN. No significant differences were found between total nitrogen and C/N ratios in lettuce cultivated in HUB‐LTE soils. Overall, lower micronutrient (Cu, Fe, Mn, Zn) contents were observed in the HUB‐LTE plants than the DOK‐LTE.

**Table 2:** Shoot and root fresh masses and nutrient content in lettuce (cv. Tizian) shoot samples after cultivation of 10 weeks in the growth chamber.

Site	Treatment (fertilization)	Shoot fresh mass [g/plant]	Root fresh mass [g/plant]	C total [g/Kg]	N total [g/Kg]	C/N	P [g/Kg]	K [g/Kg]	Na [g/Kg]	Ca [g/Kg]	Mg [g/Kg]	S [g/Kg]	Cu [p.p.m]	Fe [p.p.m]	Mn [p.p.m]	Zn [p.p.m]
DOK	CONMIN (mineral)	46.46 ± 2.55a	31.20 ± 3.17a	425.11 ± 1.57a	18.85 ± 0.48a	22.67 ± 0.62b	1.41 ± 0.05a	9.65 ± 0.77a	7.74 ± 0.26a	11.61 ± 0.64a	3.44 ± 0.1a	2.07 ± 0.04a	7.91 ± 0.29a	127.71 ± 71.16a	75.55 ± 16.27a	37.46 ± 0.97a
	BIODYN2 (biodynamic)	49.68 ± 1.82a	25.47 ± 3.47a	422.6 ± 1.4a	15.91 ± 0.45b	26.71 ± 0.82a	1.22 ± 0.03b	10.24 ± 0.4a	5.69 ± 0.27b	9.31 ± 0.81b	2.21 ± 0.09b	1.88 ± 0.06b	6.69 ± 0.28b	103.88 ± 54.54a	31.27 ± 1.48b	32.57 ± 2.2b
HUB	HU‐min (mineral)	75.21 ± 2.91a	45.96 ± 2.42a	417.07 ± 1.24b	17.1 ± 0.71a	24.7 ± 1.11a	1.4 ± 0.06b	14.25 ± 0.46a	4.9 ± 0.18b	12.72 ± 0.61a	1.88 ± 0.11a	1.46 ± 0.07a	2.36 ± 0.19b	51.99 ± 5.7a	61.71 ± 4.24a	18.09 ± 1.18a
	HU‐org (organic)	63.29 ± 2.27b	32.32 ± 2.94b	424.23 ± 1.44a	17.03 ± 0.58a	25.12 ± 0.85a	1.93 ± 0.08a	6.92 ± 0.15b	7.02 ± 0.2a	12.92 ± 0.6a	2.12 ± 0.04a	1.56 ± 0.06a	3.39 ± 0.19a	52.14 ± 3.72a	58.19 ± 2.9a	16.7 ± 0.99a

The means (*n* = 8 for nutrient content; *n* = 16 for fresh masses) per treatment are shown along with standard errors. Different lowercase letters indicate significant differences between organic versus mineral fertilization tested separately for each long‐term experimental site by pairwise *t*‐test comparisons of least squares means.

### *Plant gene expression analysis*

**Figure 1:**
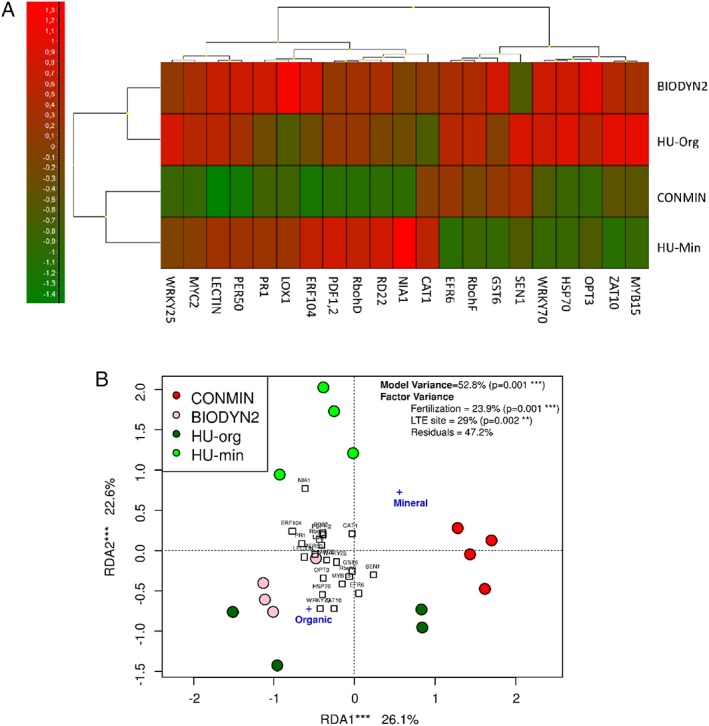
Expression of selected 21 genes in lettuce plants grown in soils of two LTE sites with different fertilization strategies [organic (BIODYN2, HU‐Org) versus mineral (CONMIN, HU‐Min)]. A. Heatmap with relative expression data. Each individual box in the heatmap represents the autoscaled ΔCt value obtained for that condition (*n* = 4). Green and red nuances correspond to lower and higher expression levels relative to the column mean respectively. Dendrograms represent hierarchical clustering between the soils (left of heatmap) or the genes (top of heatmap). B. 2D representation of RDA performed with gene expression data (ΔCt values of 21 selected genes) using fertilization type and LTE site as explanatory factors. Replicates for each treatment are colour coded and shown individually. Significance of the model, factors and axes was determined by ANOVA.

**Figure 2:**
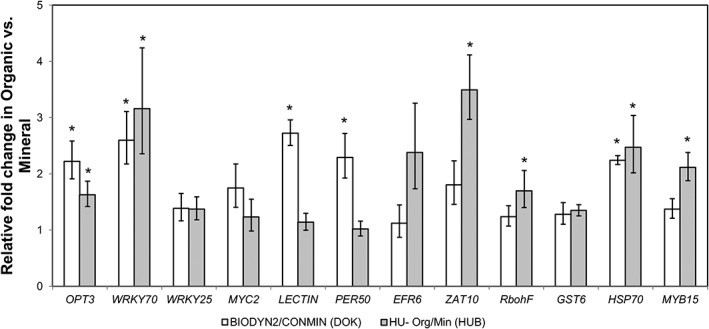
Upregulated genes in lettuce plants grown in soils with long‐term organic versus mineral fertilization (DOK‐LTE, HUB‐LTE). The 2^–ΔΔCt^ method (Kuramae *et al*., 2012) was employed for relative quantification (*n* = 4). Error bars indicate 95% confidence interval. Asterisks denote significant (p < 0.05) differences in ΔCt values between organic versus mineral fertilization within each site revealed by Tukey's HSD pairwise testing.

To emphasize how the gene expression level was influenced by the soil legacy, qPCR data were displayed in a heatmap and averaged for the biological replicates by autoscaling in gene dimension (Fig. 1A). Expression patterns of all 21 plant genes investigated grouped according to the fertilization strategy. Single gene expression patterns were organized into two distinct clusters with several subclusters. Redundancy analysis (RDA) correlation analysis showed that gene expression variability was clearly associated with tested explanatory variables. Two‐way ANOVA revealed that LTE site and fertilization strategy influenced the expression level of the selected genes and explained 29% and 23.9% of variance respectively (Fig. 1B). Although there were some differences between individual replicates, the overall expression patterns were similar from plants grown in soils with organic fertilization in both LTEs. Tukey's HSD analyses showed that several genes that encode functions involved in oxidative and biotic stress regulation pathways were differentially expressed in organic versus mineral fertilized soils. These genes were relatively upregulated in plants grown in organically fertilized soils from both LTEs (Fig. 2).

### *Analyses of prokaryotic communities in BS and lettuce rhizosphere*

In BS and rhizosphere from both sites, significantly more bacterial than archaeal 16S rRNA gene fragment copy numbers were found (p < 0.001, Fig. S1). Neither in the BS (Fig. S1A) nor in the rhizosphere (Fig. S1B), bacterial 16S rRNA gene copy numbers differed significantly between fertilization strategies. In contrast, BIODYN2 treatment resulted in significantly increased (p < 0.05) archaeal copy numbers compared with CONMIN in the rhizosphere (Fig. S1B). Chao‐1 alpha‐diversity indices and richness calculated based on 16S rRNA gene sequences were significantly reduced in the rhizosphere compared with BS except for BIODYN2 treatment (Fig. S2A and B). Calculated evenness and Shannon indices were also reduced in the rhizosphere of HUB soils compared with the BS; however, such an effect was not observed for DOK‐LTE soils (Fig. S2C and D). In both LTE sites, the fertilization strategy did not significantly influence the prokaryotic alpha‐diversity (Fig. S2).

**Figure 3:**
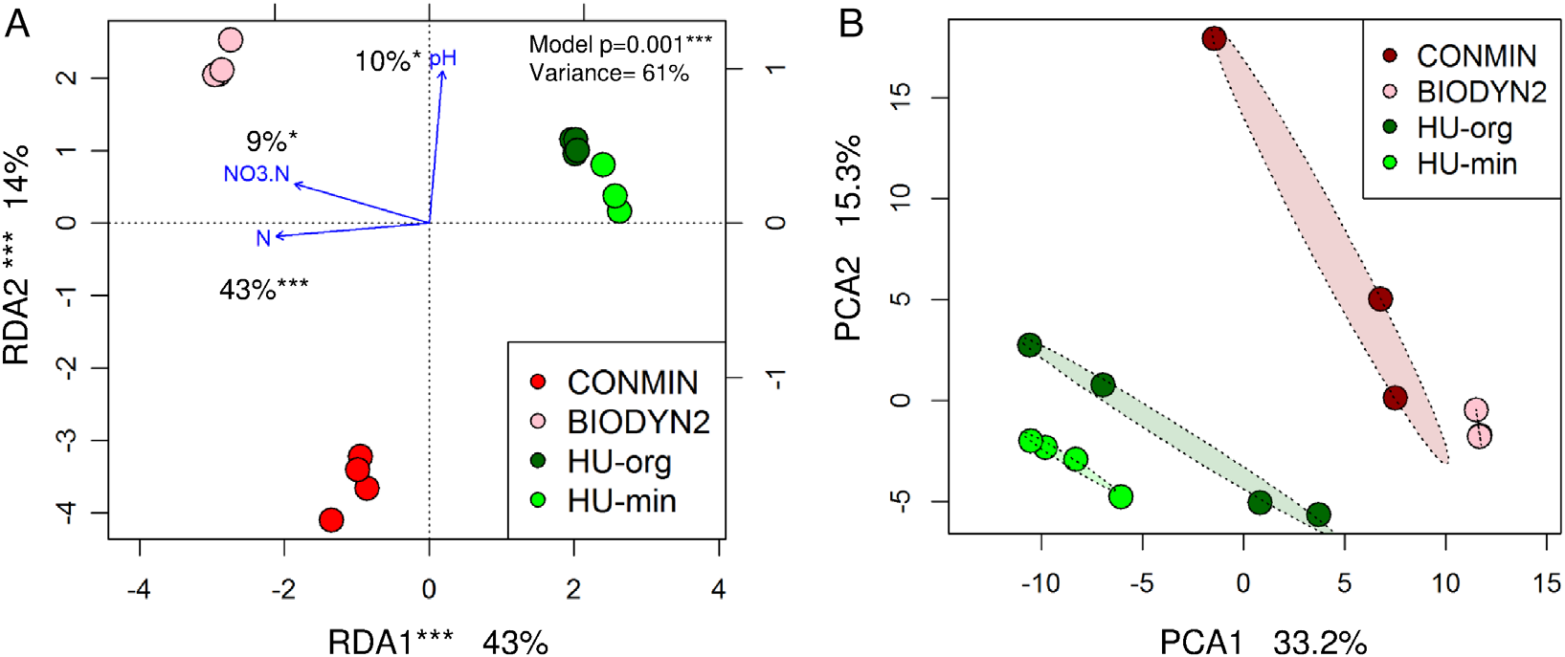
Analysis of prokaryotic communities in BS or in the rhizosphere (R) of lettuce grown in soils under long‐term organic (BIODYN2, HU‐org) or mineral (CONMIN, HU‐min) fertilization from site DOK‐LTE or HUB‐LTE, respectively, based on log‐transformed 16S rRNA gene amplicon data. A. 2D representation of RDA for BS. Nitrogen, nitrate and pH were used as explanatory factors. Significance of the model, factors and axes was determined by ANOVA. B. PCA for R.

Prokaryotic community compositions in the BS and rhizosphere differed markedly (PERMANOVA, 28% of explained variance, p < 0.001, Table S2). Therefore, the habitats were analysed separately. The site, where the soil originated from, and the fertilization strategy strongly shaped the BS prokaryotic communities (Fig. 3A). RDA explained 61% of total variance. Among included factors, nitrogen content contributed most and was associated with the first axis that separated samples according to LTE sites. The pH value, that explained 10% of variance, was related to the second axis, along which samples of different fertilization type were distributed (Fig. 3A). The structuring effect of the soil origin and fertilization was observed also in the lettuce rhizosphere (Fig. 3B). PERMANOVA confirmed that field site and fertilization influenced significantly the prokaryotic community in the rhizosphere and explained 26% or 13% of variation within the community composition respectively (Table S3). In the BS, the effect of the field site accounted for 42% and fertilization for 15% (Table S3).

Analysis at phylum and, for Proteobacteria, at class level revealed significant differences in prokaryotic community compositions between sites, especially in the BS (Fig. S3). Significantly more sequences affiliated to Thaumarchaeota and Verrucomicrobia were found in DOK‐LTE samples than HUB‐LTE. In contrast, Actinobacteria and Alpha‐, Beta‐ and Gammaproteobacteria were present in higher relative abundance in soils from HUB‐LTE. Independent of the soil origin, Firmicutes (mainly belonging to the order Bacillales) was enriched by organic fertilization resulting in a significant difference compared with mineral fertilization in the rhizosphere. Alphaproteobacteria had higher relative abundances under mineral fertilization. The prokaryotic community composition in the rhizosphere showed an enrichment of Proteobacteria (mainly of classes Alpha‐, Beta‐ and Gammaproteobacteria) and Saccharibacteria and a lower relative abundance of Acido‐ and Actinobacteria and Firmicutes compared with the BS (Fig. S3).

**Figure 4:**
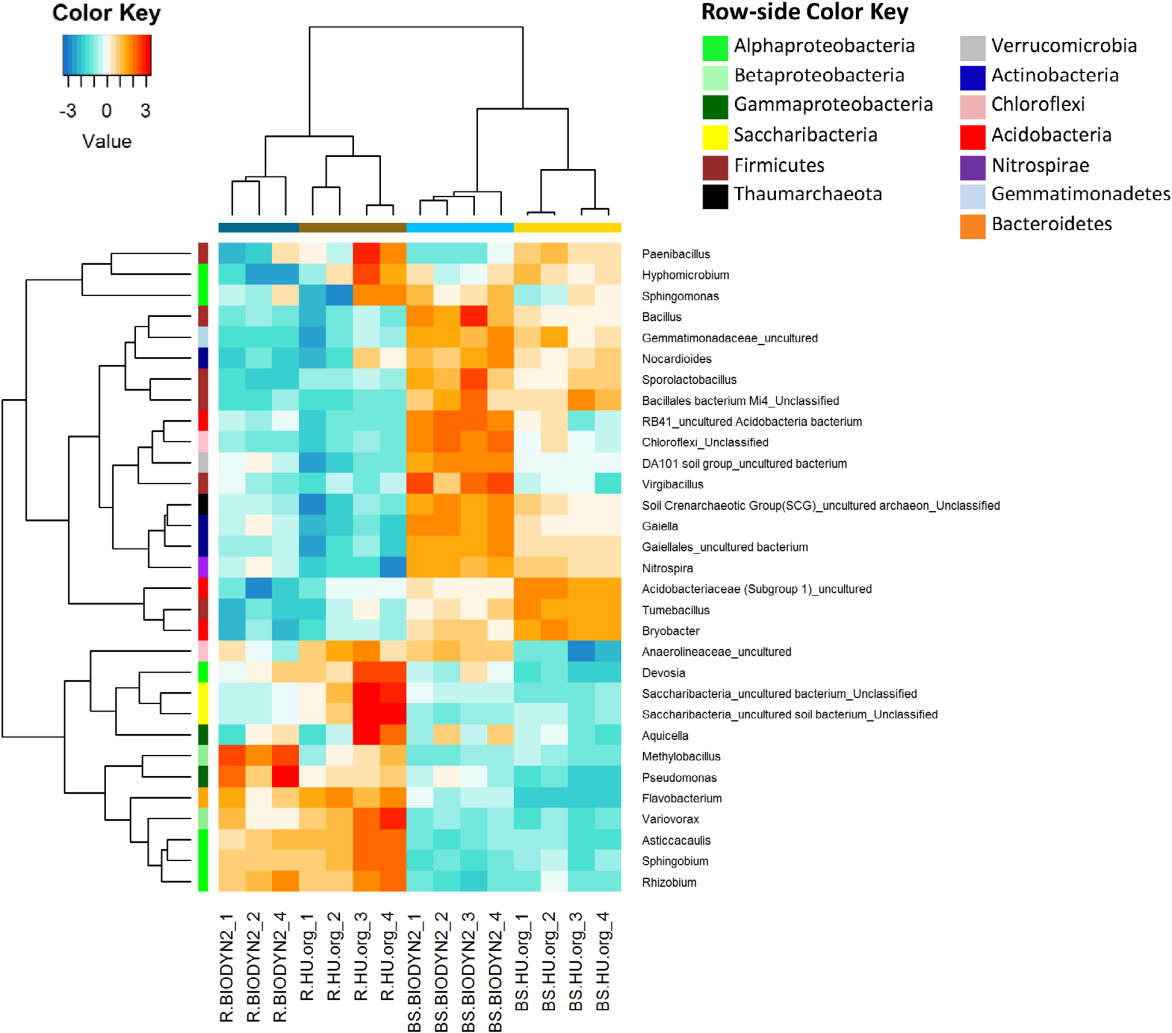
Heatmap showing the abundance of the 20 most prevalent genera that responded significantly to the rhizosphere (Likelihood ratio test, FDR‐corrected p < 0.05) in both field sites under long‐term organic fertilization (BIODYN2, HU‐org). Data were centred and scaled to the mean of each log‐transformed taxon's abundance. The column‐side colours indicate the sample type and row‐side colours indicate responder taxonomy. Nine genera belonged to significant rhizosphere responders in both field sites (see text).

Regarding the top most abundant genera, in both field sites under organic fertilization, *Rhizobium*, an unclassified genus of *Saccharibacteria*, *Asticcacaulis* and *Aquicella* were significantly enriched in the root vicinity compared with BS while *Bacillus*, *Sporolactobacillus* as well as unclassified representatives of Gemmatimonadaceae, Soil Crenarchaeotic Group, and Bacillales had a lower relative abundance in the rhizosphere (Fig. 4). However, there were also site‐dependent differences. Significantly higher relative abundances of *Flavobacterium, Variovorax, Sphingobium* and *Devosia* were observed in the rhizosphere of lettuce when grown in HU‐org. In contrast, *Pseudomonas* and *Methylobacillus* were positive rhizosphere responders (enriched in relative abundance in the rhizosphere) only in BIODYN2 soils (Fig. 4). *Rhizobium*, unclassified sequences of Saccharibacteria, and *Asticcacaulis* were found enriched in the rhizosphere of lettuce grown in soils under mineral fertilization from both sites (Fig. S4), as also observed under organic fertilization. Additionally, *Sphingobium* was found as positive rhizosphere responder of lettuce cultivated in mineral fertilized soils from both sites. In contrast, *Bryobacter* as well as unclassified members of Soil Crenarchaeotic Group and acidobacterial subgroup 1 had a lower relative abundance in the rhizosphere of both mineral fertilized soils than BS. In addition, several site‐specific rhizosphere responders were identified (Fig. S4).

Regardless of soil origin or fertilization strategy, a lettuce core rhizosphere microbiota consisting of 116 OTUs being present in all rhizosphere samples was identified (Fig. S5A). Most of the core OTUs belonged to the order Rhizobiales (27%), followed by Sphingomonadales (10%), unclassified Saccharibacteria and Bacillales (both 8%; Fig. S5B). Classification of these core OTUs on lower taxonomic levels revealed their affiliation to 65 different genera, e.g. *Rhizobium* (9%), unclassified Saccharibacteria (8%) and *Sphingomonas* (5%). Further commonly present genera were *Arthrobacter*, *Bacillus*, *Devosia*, *Mesorhizobium*, *Paenibacillus*, *Pseudomonas* and *Variovorax* (data on genus level not shown).

A number of prokaryotic genera with significantly different relative abundances in the rhizosphere of lettuce grown in mineral versus organic soils were found. The comparison of mineral or organic fertilization, respectively, among HUB‐LTE and DOK‐LTE showed several common, site‐independent fertilization responders in the rhizosphere of lettuce (Tables 3 and 4). Most of the genera with higher relative abundance in organically fertilized soils compared with mineral fertilization belonged to Firmicutes (e.g. *Thermobacillus, Planifilum, Ureibacillus*). In addition, a strong enrichment of sequences identified as *Flavobacterium* was observed in the rhizosphere of lettuce grown in both organically fertilized soils (Table 3). Mineral fertilization resulted in the enrichment of sequences belonging to *Lysobacter*. Also *Pseudoxanthomonas* was identified as positive responder to mineral fertilization in the lettuce rhizosphere (Table 4).

**Table 3:** Prokaryotic genera significantly enriched in the rhizosphere of lettuce grown in long‐term organic fertilized soils of both LTEs as compared with mineral fertilized soils according to Likelihood ratio test under negative binomial distribution and generalized linear models.

						DOK			HUB		
Domain	Phylum	Class	Order	Family	Genus	CONMIN	BIODYN2	p value	HU‐Min	HU‐Org	p Value
Archaea	Thaumarchaeota	SIGW621	Uncultured bacterium	Unclassified	*Unclassified*	0.01 ± 0.01	0.32 ± 0.15 ↑	***	0 ± 0	0.01 ± 0.01 ↑	*
Bacteria	Actinobacteria	Acidimicrobiia	Acidimicrobiales	Uncultured	*Uncultured Acidimicrobidae bacterium*	0.01 ± 0.01	0.05 ± 0.02 ↑	*	0 ± 0	0.02 ± 0.01 ↑	**
Bacteria	Actinobacteria	Actinobacteria	Streptosporangiales	Streptosporangiaceae	*Thermopolyspora*	0 ± 0	0.03 ± 0.01 ↑	**	0 ± 0	0.01 ± 0.01 ↑	**
Bacteria	Bacteroidetes	Cytophagia	Cytophagales	Cytophagaceae	*Ohtaekwangia*	0.02 ± 0.03	0.63 ± 0.37 ↑	***	0 ± 0	0.02 ± 0.02 ↑	**
Bacteria	Bacteroidetes	Flavobacteriia	Flavobacteriales	Flavobacteriaceae	*Flavobacterium*	0.05 ± 0.02	1.36 ± 1.36 ↑	***	0.49 ± 0.77	2.53 ± 2.06 ↑	*
Bacteria	Firmicutes	Bacilli	Bacillales	Thermoactinomycetaceae	*Thermoflavimicrobium*	0 ± 0	0.31 ± 0.16 ↑	***	0.02 ± 0.01	0.05 ± 0.02 ↑	*
Bacteria	Firmicutes	Bacilli	Bacillales	Paenibacillaceae	*Thermobacillus*	0 ± 0	0.07 ± 0.01 ↑	***	0.01 ± 0.01	0.1 ± 0.04 ↑	***
Bacteria	Firmicutes	Bacilli	Bacillales	Thermoactinomycetaceae	*Planifilum*	0 ± 0	0.05 ± 0.02 ↑	***	0.01 ± 0.01	0.04 ± 0.03 ↑	*
Bacteria	Firmicutes	Bacilli	Bacillales	Family XII	*Incertae Sedis*	0 ± 0	0.02 ± 0 ↑	**	0 ± 0	0.01 ± 0.01 ↑	**
Bacteria	Firmicutes	Bacilli	Bacillales	Bacillaceae	*Ureibacillus*	0.01 ± 0.01	0.04 ± 0.05 ↑	*	0 ± 0.01	0.11 ± 0.06 ↑	***
Bacteria	Firmicutes	Erysipelotrichia	Erysipelotrichales	Erysipelotrichaceae	*Asteroleplasma*	0.03 ± 0.03	0.15 ± 0.07 ↑	*	0 ± 0	0.04 ± 0.04 ↑	***
Bacteria	Proteobacteria	Deltaproteobacteria	Bdellovibrionales	Bdellovibrionaceae	*Bdellovibrio*	0.02 ± 0.02	0.18 ± 0.11 ↑	**	0.18 ± 0.09	0.42 ± 0.23 ↑	*
Bacteria	Verrucomicrobia	Verrucomicrobiae	Verrucomicrobiales	Verrucomicrobiaceae	*Roseimicrobium*	0.01 ± 0.02	0.09 ± 0.04 ↑	**	0.01 ± 0.01	0.18 ± 0.3 ↑	***

The average relative abundances (*n* = 4, except CONMIN and BIODYN2: *n* = 3) are shown along with standard deviation.

*p < 0.05; **p < 0.01; ***p < 0.001.

**Table 4:** Prokaryotic genera significantly enriched in the rhizosphere of lettuce grown in long‐term mineral fertilized soils of both LTEs as compared with organic fertilized soils according to Likelihood ratio test under negative binomial distribution and generalized linear models.

						DOK			HUB		
Domain	Phylum	Class	Order	Family	Genus	CONMIN	BIODYN2	P Value	HU‐Min	HU‐Org	P Value
Bacteria	Actinobacteria	Actinobacteria	Micrococcales	Intrasporangiaceae	*Lapillicoccus*	0.5 ± 0.24 ↑	0.1 ± 0.08	***	0.81 ± 0.31 ↑	0.48 ± 0.14	*
Bacteria	Actinobacteria	Actinobacteria	Micrococcales	Microbacteriaceae	*Lysinimonas*	0.3 ± 0.12 ↑	0.09 ± 0.05	***	0.66 ± 0.2 ↑	0.25 ± 0.11	**
Bacteria	Chloroflexi	Ktedonobacteria	C0119	Uncultured Chloroflexi bacterium	*Unclassified*	0.13 ± 0.1 ↑	0 ± 0	***	0.13 ± 0.05 ↑	0.03 ± 0.03	***
Bacteria	Chloroflexi	Ktedonobacteria	Ktedonobacterales	Ktedonobacteraceae	*Uncultured*	0.04 ± 0.05 ↑	0.01 ± 0.01	*	0.09 ± 0.05 ↑	0.04 ± 0.03	*
Bacteria	Proteobacteria	Gammaproteobacteria	Xanthomonadales	Xanthomonadaceae	*Pseudoxanthomonas*	0.65 ± 0.29 ↑	0.16 ± 0.04	***	2.53 ± 0.61 ↑	0.55 ± 0.27	***
Bacteria	Proteobacteria	Gammaproteobacteria	Xanthomonadales	Xanthomonadaceae	*Lysobacter*	0.4 ± 0.25 ↑	0.25 ± 0.11	*	1.65 ± 1.84 ↑	0.2 ± 0.09	***

The average relative abundances (*n* = 4, except CONMIN and BIODYN2: *n* = 3) are shown along with standard deviation.

*p < 0.05; **p < 0.01; ***p < 0.001.

## Discussion

Using a multidisciplinary approach coupling soil properties together with RNA‐ and DNA‐based approaches, we explored the effect of long‐term fertilization strategies and field site context on the rhizosphere microbiota assemblage and performance of lettuce. Lettuce was chosen as model plant due to its sensitivity towards abiotic stress factors and since it was never cultivated in these soils before. Our experimental design in a growth chamber allowed us to investigate how lettuce growth and health were influenced by the biotic and abiotic soil properties conditioned in the long term by different fertilization strategies at two different field sites.

### *Plant‐driven selection of rhizosphere prokaryotic community assemblage*

The experiment was conducted with soils originating from two different sites with long‐term organic and mineral fertilization strategies. Consistent with previous studies (Bulgarelli *et al*., 2012; Kuramae *et al*., 2012; Schreiter *et al*., 2014), we observed that each soil type, exhibiting different physicochemical properties, had a unique soil prokaryotic community (Fig. 3A). The DOK‐LTE is a well‐described and matured experimental system, which aims to compare the long‐term effects of conventional versus organic management strategies on agroecosystem properties (Mäder *et al*., 2002). Recently, next‐generation sequencing revealed that long‐term agricultural farming strategies distinctly shaped soil microbial community structures at DOK‐LTE (Hartmann *et al*., 2015). The dominant phyla found in our study in the DOK‐LTE BSs and the prevalence of Firmicutes in BIODYN2 compared with CONMIN soils corresponded to results of Hartmann *et al*. (2015). In contrast, the prokaryotic community composition at HUB‐LTE has not been studied before.

As expected, we observed in all treatments a strong shift of the prokaryotic community in the vicinity to lettuce roots (Fig. S3) due to rhizodeposition that serve many soil microorganisms as nutrient source. It is well known that root exudates allow plant species to selectively recruit microorganisms from the soil environment (Bais *et al*., 2006; Bakker *et al*., 2013) that might be beneficial for their growth, fitness and health (Mendes *et al*., 2013; Philippot *et al*., 2013). In a previous study, the soil type‐independent enrichment of several genera in the rhizosphere of lettuce was shown (Schreiter *et al*., 2014). Moreover, it has been demonstrated that the rhizosphere microbiota of *Arabidopsis thaliana* is partially shared across different soil types and plant developmental stages suggesting the existence of a core microbiota (Lundberg *et al*., 2012). In congruence with Schreiter *et al*. (2014), we could show the existence of a lettuce rhizosphere core microbiota (e.g. *Sphingomonas*, *Rhizobium*, *Variovorax*, Saccharibacteria) that was present independently of the site the soil originated from and the long‐term fertilization strategy (Fig. S5).

However, besides the core rhizosphere microbiota, there is evidence that external factors individualize community assemblages (Bulgarelli *et al*., 2012; Lundberg *et al*., 2012, Schöps *et al*., 2018). Confirming our hypothesis, we observed that the rhizosphere prokaryotic community was significantly affected by the fertilization strategy, contributing about 20%–43% to the OTU presence (Fig. S5A). Despite intensive studies on long‐term fertilization effects on the BS microbiota, little is known on how this impacts the rhizosphere microbiota assemblage and possibly the aboveground plant performance. Although there was no significant difference between the fresh mass of the plants in relation to the type of fertilization history within the DOK site, plants had more biomass when grown on mineral than organically fertilized soils from HUB‐LTE (Table 2). Interestingly, when comparing the expression of selected genes on lettuce, similar patterns were observed depending on the fertilization strategy across different LTE sites despite different soil characteristics, maturity of the experimental systems and last crops in the field (Fig. 1). Therefore, we focused on revealing common fertilization‐dependent trends in the rhizosphere prokaryotic communities, which could help to explain plant gene expression patterns arising from interactions with the prokaryotes at the roots. We compared responders (prokaryotic taxa) that were enriched in the rhizosphere of lettuce cultivated in soils with either organic or mineral fertilization history independent of the LTE sites (Tables 3 and 4). Most genera with increased relative abundance in the rhizosphere of lettuce grown in organically fertilized soils belonged to the phylum Firmicutes. Rhizobacteria of this phylum have been widely studied and suggested to belong to the ‘good’ (i.e. plant‐beneficial) proportion of rhizosphere bacteria (Mendes *et al*., 2013). We observed mainly an increase in Firmicutes sequences classified as Bacillales and not of Clostridiales, the latter being characteristically fermenting and often originating from organic fertilizers (Wolters *et al*., 2018). Therefore, we suggest that indigenous soil bacteria affiliated to Firmicutes were fostered by the type of nutrients added with the organic fertilizer. An increase in Bacillales in organically fertilized soils was reported before (Schmid *et al*., 2017). Furthermore, OTUs affiliated to the genus *Flavobacterium* were enriched in the rhizosphere of lettuce grown in both organically fertilized soils (BIODYN2, HU‐org) in the present study (Table 3). Species of this genus are well adapted to the plant carbon metabolism, which might have led to their enrichment in the rhizosphere (Kolton *et al*., 2013). Firmicutes and *Flavobacterium* were frequently associated with disease suppressive soils (Mendes *et al*., 2013, Gómez Expósito *et al*., 2017 and references therein). Long‐term mineral fertilization on the other hand resulted in a higher relative abundance of *Lysobacter* and *Pseudoxanthomonas* sequences in the rhizosphere of lettuce (Table 4). The genus *Lysobacter* comprises strains that were isolated from suppressive soils and showed biocontrol activity against phytopathogens (Gómez Expósito *et al*., 2017). Thus, the rhizosphere prokaryotic communities from both organically and mineral fertilized soils showed an enrichment in members with potential plant‐beneficial and suppressive characteristics towards soil‐borne plant pathogens. This observation can probably be explained by the concept of functional microbial redundancy in soil (Griffiths *et al*., 2000), which proposes that diverse soil microbial communities in terms of species richness and evenness can compensate the effects of environmental fluctuations in the long term by carrying out similar functions insuring maintenance of ecosystem processes. In the present study, the legacy of long‐term organic or mineral fertilization affected the prokaryotic community structure yet not alpha‐diversity measures in the respective soils. Irrespective of fertilization history and field site, we observed a plant‐driven selection from BS and an enrichment of populations with likely redundant plant‐associative traits in the rhizosphere, which may have assisted the observed proper development of lettuce plants in these different soils.

### *Plant gene expression and belowground interactions*

Analyses of the gene expression levels revealed that several genes with roles in response to stress or biotic stimuli were up‐regulated in plants grown in soils with organic compared with mineral fertilization history (Fig. 2). In the lettuce leaves from organically fertilized soils, we observed higher transcription levels of *PER50*, *ERF6*, *RbohF*. These genes are involved in responses to intracellular oxidative stress and/or pathogenesis in *Arabidopsis* (Chaouch *et al*., 2012; Dubois *et al*., 2015). However, redox homeostasis and reactive oxygen species (ROS) production are associated with several biological processes in plants and the up‐regulation of some gene families involved in oxidative stress signalling may also indicate enhanced tolerance to abiotic and biotic stresses (Miller *et al*., 2008). Increased oxidative activity in leaves can also occur during enhanced leaf growth and photosynthetic activity (Hideg and Schreiber, 2007), which has been shown to be augmented by the presence of active plant growth promoting rhizobacteria (PGPR) (Carvalhais *et al*., 2013). The here observed enhanced expression of the zinc‐finger transcription factor *ZAT10*, which plays a key role in abiotic stress tolerance (Mittler *et al*., 2006), and *MYB15*, a member of the R2R3 MYB family of transcription factors in *Arabidopsis* reported to be upregulated by (a)biotic stresses (Liu *et al*., 2015), indicates an enhanced tolerance to stress in these plants. A higher expression of the selected (Oxylipin) OPDA‐dependent, jasmonic acid (JA)‐independent genes encoding glutathione S transferase 6 (*GST6*), and heat shock protein 70 (*HSP70*) was also observed in leaves of plants pretreated with oxo‐C14‐HSL showing induction of systemic resistance by the quorum sensing molecules N‐Acyl homoserine lactones (AHLs) (Schenk *et al*., 2014). Induced resistance in healthy tissues can occur by long‐distance signalling due to pathogen‐induced resistance, commonly known as systemic acquired resistance (SAR) distal from the site of infection (Spoel and Dong 2012) as well as due to ISR by beneficial microbes (van Loon *et al*., 2006; Pieterse *et al*., 2014). Therefore, the observed induction of several genes involved in oxidative stress, SA‐ (*WRKY70, WRKY25*), JA‐ (*LECTIN, MYC2*) ISR and ethylene response factors in shoots may be an overall result of defence reactions due to encounters that are continuously occurring during plant growth with MAMPs of PGPR as well as PAMPs (microbe‐associated/pathogen‐associated molecular patterns) and effectors of pathogens in the rhizosphere. The fact that the observed responses occurred in shoots indicates their systemic rather than local induction and may be a combination of belowground interactions of the plant roots with a multitude of microbes in the rhizosphere. The increased expression of these genes in healthy plants indicates an induced physiological status that can result in an enhanced resistance to biotic stresses or higher tolerance to abiotic stresses (defence priming) (Martinez‐Medina *et al*., 2016). Our observations are further supported by the upregulation of the *OPT3* gene, a key component of Fe signalling network (Zhai *et al*., 2014; Khan *et al*., 2018) indicating deficiency in bio‐available Fe. It has been recently proposed that rhizosphere microbes can induce *MYB72*/*BGLU42‐*dependent ISR response *via* iron‐mobilizing phenolics, simulating root iron‐deficiency response and changes in iron‐homeostasis mechanisms in the rhizosphere, which can be expressed systemically throughout the plant (Verbon *et al*., 2017; Stringlis *et al*., 2018). In addition, it is assumed that the plant immunity has co‐evolved with the plant microbiome and thus plays an essential role in determining the structure of the plant‐associated microbiota (Turner *et al*., 2013). In contrast to the challenge of plants with phytopathogens, beneficial bacteria modulate the expression of plant‐defence‐related genes differently and at a lower level (Brusamarello‐Santos *et al*., 2012). We suppose that the enrichment of Bacillales and other taxa previously associated with disease‐suppressive soils (see above) in the rhizosphere of lettuce grown in organically fertilized soils could contribute to such an induced state in the plants. Nevertheless, the primed state is often undetectable in unchallenged plants (Pieterse *et al*., 2014). Further studies with challenging pathogens are needed to reveal whether lettuce grown in organically fertilized soils is better prepared for a pathogen attack compared with mineral fertilization.

### *Positive aboveground response observed for model plant lettuce*

Owing to the complexity of the effects of agricultural management on the soil microbiota, making universally valid conclusions on organic and mineral fertilization strategies is difficult. It has been reported that low‐input organic farming systems not relying on agrochemicals usually promote higher abundance and diversity of most organisms (Bender *et al*., 2016). However, this could not be confirmed in our study neither for prokaryotic community diversity in BS nor in the lettuce rhizosphere. In this study, the plants grown in different soils had a comparable biomass and were free from visible disease symptoms. Moreover, taxa with close similarity to plant growth promoting bacteria were found in the rhizosphere of lettuce irrespective of the long‐term fertilization and field site (Tables 3 and 4). Under the given experimental conditions, we therefore observed a positive belowground/aboveground plant–microbe interaction for soils from both field sites and both fertilization strategies. However, as stated above, we suggest an induced physiological status of lettuce when grown in long‐term organically fertilized soils. With our experimental setup, we cannot ultimately clarify the underlying causal relationship for the belowground/aboveground interaction. Therefore, further studies such as cross‐inoculation experiments with sterile soils under controlled conditions are needed. In addition, it remains to be elaborated to which extent our results can be extrapolated to conditions in the fields where, e.g. plant competition, nutrient availability and aboveground biotic interactions can play important roles (van der Putten *et al*., 2016).

Taken together, our results showed that prokaryotic community structures in the soil are shaped by the long‐term fertilization strategy that in turn impacted the rhizosphere microbiota assemblage as observed by the enrichment of different sets of genera in the rhizosphere in response to mineral and organic long‐term fertilization regimes. The observed core rhizosphere microbiota shared across plants cultivated in the soils of different field site origin and fertilization history indicate towards a plant‐driven selection and enrichment of bacterial populations with likely plant‐associative traits. The redundancy of plant‐associative traits in the enriched rhizosphere microbiota and the establishment of a core microbiota of lettuce (which was never cultivated in these soils) irrespective of fertilization history and field site, together with plant growth and shoot gene expression evidences, indicate a plant‐driven selection and a particular rhizosphere microbiota assemblage to ensure proper plant development. The potential to improve the sustainability of farming strategies through management of the rhizosphere microbiota (“soil biological engineering”) has been recently proposed (Bender *et al*., 2016). Our results add to this concept by providing insights into the crucial role of the plant in driving rhizosphere microbiota assemblage as well as influencing aboveground/belowground plant–microbe interactions, and resulting feedback mechanisms increasing plant performance. Future experiments planned with field grown crops will contribute towards the understanding of belowground/aboveground interactions and plant–soil feedbacks occurring in agroecosystems.

## Experimental procedures

### *Study sites and soil sampling strategy*

One LTE study site was located in Thyrow, Germany (52°16’N, 13°12′E; Ellmer, and Baumecker, 2016) established in 2006 by Humboldt University Berlin (HUB‐LTE) with the soil being classified as an Albic Luvisol. The other LTE site was located in Therwil, Switzerland (47°30’N, 7°33′E; Mäder *et al*., 2002) established in 1978 by the Research Institute of Organic Agriculture (FIBL; DOK‐LTE) and the soil is designated as a Haplic Luvisol. In both LTEs, soils were collected in 2015 after harvest of the standing crops from fields with two different types of long‐term fertilization strategies [mineral (CONMIN, HU‐min) versus organic (BIODYN2, HU‐org)], with four independent replicates each (Table S1). Samples were taken from the upper 30‐cm soil horizons as a combined sample of 15 soil cores randomly distributed across the plot area. After sampling, soil was air‐dried, sieved (4‐mm mesh) and stored in the dark at 6°C, 80% relative humidity (RH) until the growth chamber experiment was started.

### *Design of growth chamber experiment and sampling*

Before sowing of lettuce, soils were incubated for 2 weeks in the dark (20°C day/15°C night, 60% RH/80% RH) with 100‐hPa water potential (T5 tensiometer, UMS AG, Munich, Germany). Lettuce (*Lactuca sativa* var. *capitata* cv. ‘Tizian’, Syngenta, Bad Salzuflen, Germany) was germinated in the soils from the two LTEs (mineral versus organic) and seedlings at BBCH13 to BBCH14 growth stage were transferred individually into pots (10 cm × 10 cm × 11 cm) containing the respective soils (Table S1). To ensure that each plant in each treatment had comparable amounts of nitrogen available, the N contents of soils were measured before planting. Soils were fertilized using calcium nitrate to 50% of the recommended amounts of N for lettuce growth (0.32 g/pot) during planting of seedlings and the remaining amount was added after 2 weeks. Each treatment included four replicates with four plants per replicate. All pots were placed in a growth chamber in randomized block design. Lettuce plants were cultivated at 20°C day/15°C night, 60% RH/80% RH, 16 h day with 420 μmol m^−2^ s^−1^ photosynthetic active radiation and 100‐hPa water potential. The plants were harvested after 10 weeks at BBCH19 growth stage. Shoot and root fresh masses were analysed with a one‐way ANOVA via SAS procedure ‘MIXED’ (http://www.sas.com/) followed by Tukey's HSD tests considering heteroscedasticity by using group variances. The content of macro‐ and micronutrient in soil and plant samples were analysed according to the certified protocols of Agricultural Analytic and Research Institutions Association (VDLUFA, Germany). Content of K, Na, P, Mg, Fe, Mn, Cu and Zn in soils and lettuce shoots was measured via inductively coupled plasma optical emission spectrometry (ICP‐OES); C total, N total and NO3—N was analysed via elemental analysis (FIAmodula); soil pH was determined via calcium chloride solution and Salt via electrical conductivity. Differences in shoot and root fresh masses as well as nutrient contents between organic versus mineral fertilization were analysed separately for each long‐term experimental site by pairwise *t*‐test comparisons. For rhizosphere prokaryotic community analysis, complete roots of two plants per replicate were pooled to a composite sample. Microbial cells were recovered from 5‐g roots by Stomacher treatment followed by centrifugation according to Schreiter *et al*. (2014). BSs that were incubated under same conditions but without lettuce plants, and rhizosphere pellets were stored at −20°C until total microbial community (TC)‐DNA extraction.

### *Plant gene expression studies*

Target genes of lettuce were selected based on comparisons with functional genes from *A. thaliana* involved in abiotic or biotic stress pathways using ‘The Arabidopsis Information Resource’ (http://www.arabidopsis.org, Berardini *et al*., 2015). The complete sequences of the corresponding 21 genes (Table S4) were obtained from the *Lactuca sativa* whole genome shotgun sequencing project at NCBI (http://www.ncbi.nlm.nih.gov/bioproject/PRJNA68025). The reference gene glyceraldehyde‐3‐dehydrogenase was used as an endogenous control in qPCR analyses (Chowdhury *et al*., 2015). The primer pairs for qPCR (Table S4) were designed with the Primer3Plus software (Untergasser *et al*., 2007). The target and endogenous control genes were validated and only primers with 100% (±10%) efficiencies were used (Applied Biosystem Real‐time PCR handbook guidelines, Thermo Fisher Scientific). The primer/gene‐specificities were checked by PCR on cDNAs and the amplicon sequences were aligned to the *Lactuca sativa* whole genome using BLAST (Altschul *et al*., 1997).

Four leaves per plant from outer to inner whorls were pooled and snap frozen in liquid nitrogen. Pulverized leaves of 100 mg were subjected to RNA extraction using the RNeasy Plant Mini Kit (QIAGEN GmbH, Hilden, Germany). RNA was quantified by a NanoDrop spectrophotometer (Thermo Fisher Scientific, Waltham, MA, USA). cDNA was synthesized from 2 μg of RNA with the High Capacity cDNA Reverse Transcription Kit with RNase Inhibitor (Applied Biosystems, Foster City, CA, USA). qPCR was performed as described previously (Chowdhury *et al*., 2015) in quadruplicates with two plants for each replicate. The generation of specific PCR products was confirmed by melting curve analysis and gel electrophoresis. The 2^–ΔΔCt^ method (Livak and Schmittgen 2001) was employed for relative quantification. Normalization to the endogenous control for each condition was followed by logarithmic transformation to fold‐change differences. The standard error of the mean was calculated from the average of technical triplicates, obtained from each of four biological replicates.

The software GenEx (http://www.multid.se/) was used for analysis and visualization. The data were scaled either using mean centring or autoscaling (Bergkvist *et al*., 2010). Hierarchical clustering was performed by Ward's algorithm. The expression matrix was visualized by a colour‐coded heatmap. To compare which genes were differentially expressed in organic versus mineral fertilization, one‐way ANOVA with post‐hoc Tukey's HSD tests were performed. RDA was carried out in R3.1.3 (https://www.r-project.org/) with package *vegan* based on ΔCt data using fertilization type and LTE site as constraint variables. The significance of the model, RDA axis and factors were tested by ANOVA.

### *Prokaryotic community analyses*

TC‐DNA was extracted from BS (0.5 g) and rhizosphere pellets using the FastPrep‐24 bead‐beating system and FastDNA Spin Kit for Soil and subsequently purified with the GeneClean Spin Kit (both MP Biomedicals, Santa Ana, CA, USA). No TC‐DNA was obtained for one replicate of CONMIN rhizosphere (CONMIN‐1). Prokaryotic communities were characterized based on sequencing of the V3‐V4 region of 16S rRNA genes amplified using primer pair 341F (Sundberg *et al*., 2013) and 806R (Caporaso *et al*., 2011) targeting both *Bacteria* and *Archaea* kingdoms (Table S5). PCRs were performed in 25‐μl volumes containing 0.625 U Taq Polymerase (TrueStart, Thermo Scientific, Waltham, MA, USA), 0.2 mM of each dNTP, 2.5 mM MgCl_2_, 0.3 μM of each primer and 1 μl of target DNA. PCR conditions were as follows: 2 min at 94°C, 30 cycles of 20 s at 94°C, 20 s at 56°C, 40 s at 72°C, followed by a final elongation step for 5 min at 72°C. Tagging and adding of sequencing adapters was performed as previously described (Nunes *et al*., 2016). Purification and size‐selection (removal of products <200 bp) was performed using Agencourt AMPure XP beads (Beckman Coulter, Brea, CA, USA). Samples were pooled and adjusted to equimolar amounts and concentrated using the DNA Clean and Concentrator‐5 kit (Zymo Research, Irvine, CA). High‐throughput amplicon sequencing of 16S rRNA genes [2 × 250 bp, paired‐end, Illumina MiSeq platform (Illumina, San Diego, CA, USA)] was carried out following manufacturer's instructions. Both, sequencing and analysis, were done according to acknowledged best practice guidelines (Schöler *et al*., 2017; Jacquiod *et al*., 2018). Unassembled raw amplicon data was submitted to NCBI Sequence Read Archive (SRA, https://www.ncbi.nlm.nih.gov/sra) under accession number SRP133289.

Sequences were processed as described in Nunes *et al*., 2016. Briefly, after demultiplexing and trimming, sequences were paired and filtered (maxee 0.5) with *usearch* v7.0.1090 (Edgar, 2010). Removal of singletons and clustering to operational taxonomic units (OTUs, >97% sequence similarity) was carried out with UPARSE (Edgar, 2013). *Usearch* and the *ChimeraSlayer* package (Haas *et al*., 2011) were applied for chimera filtering. Representative OTUs were classified using SILVA123.1 SSURef database. Sequences affiliated to Cyanobacteria/chloroplasts, mitochondria or unclassified at the domain level were discarded, resulting in a total of 5956 OTUs. The rhizosphere sample BIODYN2‐3 and BS sample HU‐min‐4 were removed from the data set due to low amounts of sequences. On average, 12,433 quality‐filtered sequences per sample were obtained. Multivariate analysis was carried out by R3.1.3 (packages *edgeR, vegan, phyloseq, VennDiagram, gplots, RColorBrewer, rioja, mvabund*). Alpha‐diversity indices (richness, Shannon, Chao, evenness) were calculated for 100 times randomly, to the lowest amount of sequences subsampled data set (3209). Average of indices per replicate were computed and tested for differences using pairwise *t*‐test (p < 0.05). To compare prokaryotic community compositions between samples of different habitat, fertilization and soil site, PERMANOVA (Euclidean distance, 10,000 permutations) and principal component analysis (PCA) were executed on log_10_ transformed data (not subsampled). Log_10_‐transformed BS data were subjected to RDA as described above using nitrate, nitrogen and pH as explanatory factors (Table 1). For each fertilization type separately, genera with significantly different relative abundances between rhizosphere and BS were tested by Likelihood ratios under negative binomial distribution and generalized linear models with data normalization (*edgeR*; FDR‐corrected p < 0.05; considering only genera present in at least three replicates out of four). Out of these, the 20 most abundant genera from both soil sites were selected and their abundance graphically displayed in a heatmap based on centred‐scaled log_10_‐transformed counts for each taxon. Similar testing schemes were applied to identify genera with significantly different relative abundances between fertilization treatments at each soil site. To assign genera that were enriched in organic or mineral fertilization at both soil sites, significant positive responders (p < 0.05) of, e.g. CONMIN (DOK‐LTE) and HU‐min (HUB‐LTE) were compared. The rhizosphere core microbiota were visualized by Venn diagrams with OTUs present in all replicates of each treatment.

TaqMan‐based approaches were used to quantify bacterial and archaeal 16S rRNA genes (Table S5). qPCR for bacterial and archaeal 16S rRNA genes was conducted as described previously (Vogel *et al*., 2014). Log_10_‐transformed data were related to soil or root dry weight, respectively, and tested for significance using three‐way ANOVA (organism, soil site, fertilization) followed by pairwise *t*‐test comparisons in R.

## Supporting information


**Appendix S1:** Supporting information.
