# Supplementary material for: Effect of long‐term organic and mineral fertilization strategies on rhizosphere microbiota assemblage and performance of lettuce
Source: Environ Microbiol. 2019 Apr 29;21(7):2426–39. doi: 10.1111/1462-2920.14631 (PMC6849853; doi:10.1111/1462-2920.14631)
Supplement: Supplementary file 1 — Appendix S1: Supporting information. [file EMI-21-2426-s001.doc]

**Supplementary figures and tables**

**
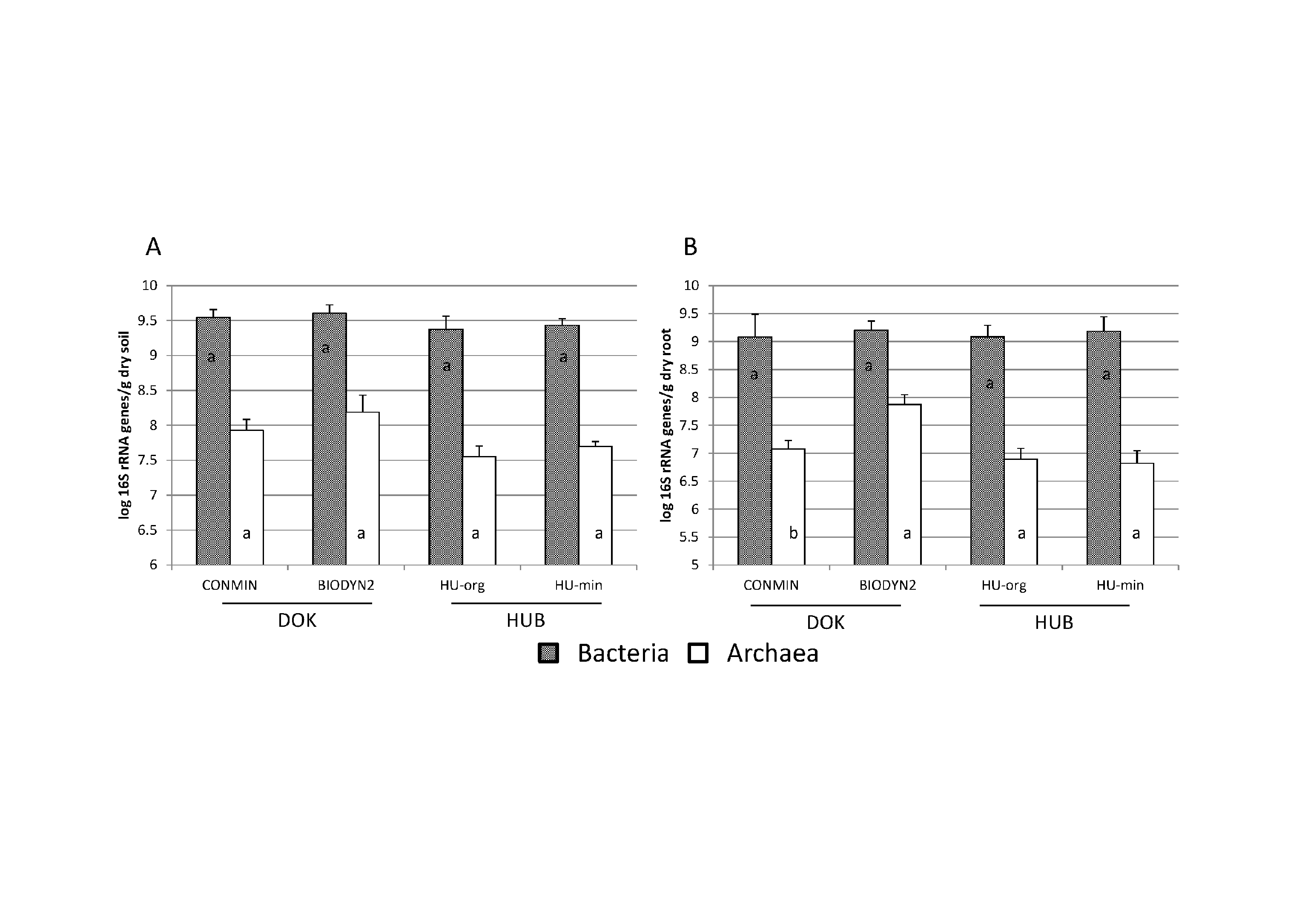
**

Figure S1: Quantification of bacterial and archaeal 16S rRNA gene fragments by qPCR, A) bulk soil and B) rhizosphere of lettuce grown in soils under long-term organic (BIODYN2, HU-org) or mineral (CONMIN, HU-min) fertilization from site DOK-LTE or HUB-LTE, respectively. Average values along with standard deviations are shown (n=4; except CONMIN rhizosphere n=3). Data was tested for significance by three-way ANOVA which revealed a significant effect of the organism type (p<0.001). Therefore, effect of fertilization strategy was tested separately for each site and organism type by pairwise t-test comparisons. Significant differences are displayed by different lowercase letters (p<0.05).


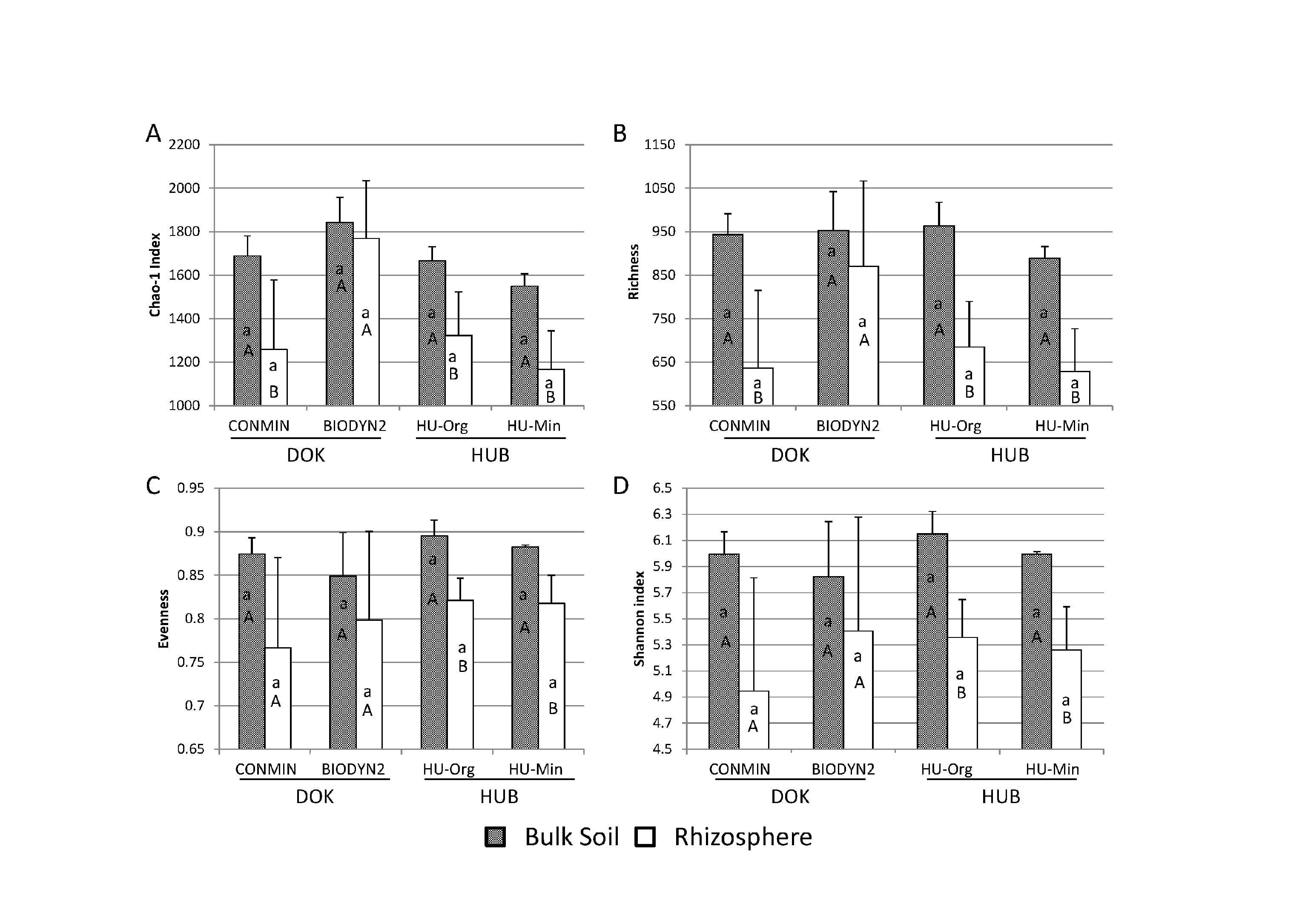
Figure S2: Prokaryotic alpha-diversity in bulk soil (BS) and rhizosphere (R) of lettuce grown in organic or mineral fertilized soils from site DOK-LTE or HUB-LTE, respectively. A- Chao-1 index, B- Richness, C- Pielou’s Evenness, D- Shannon Index. All calculations are based on 100 times randomly subsampled 16S rRNA gene amplicon data. Average values along with standard deviations are shown (n=4; except CONMIN and BIODYN2 rhizosphere, HU-min bulk soil n=3). Different lowercase letters indicate significant differences between Org *vs.* Min tested separately for each site and habitat, different capital letters denote significant differences between R *vs.* BS tested separately for each site and treatment based on pairwise t-test comparisons (p<0.05).


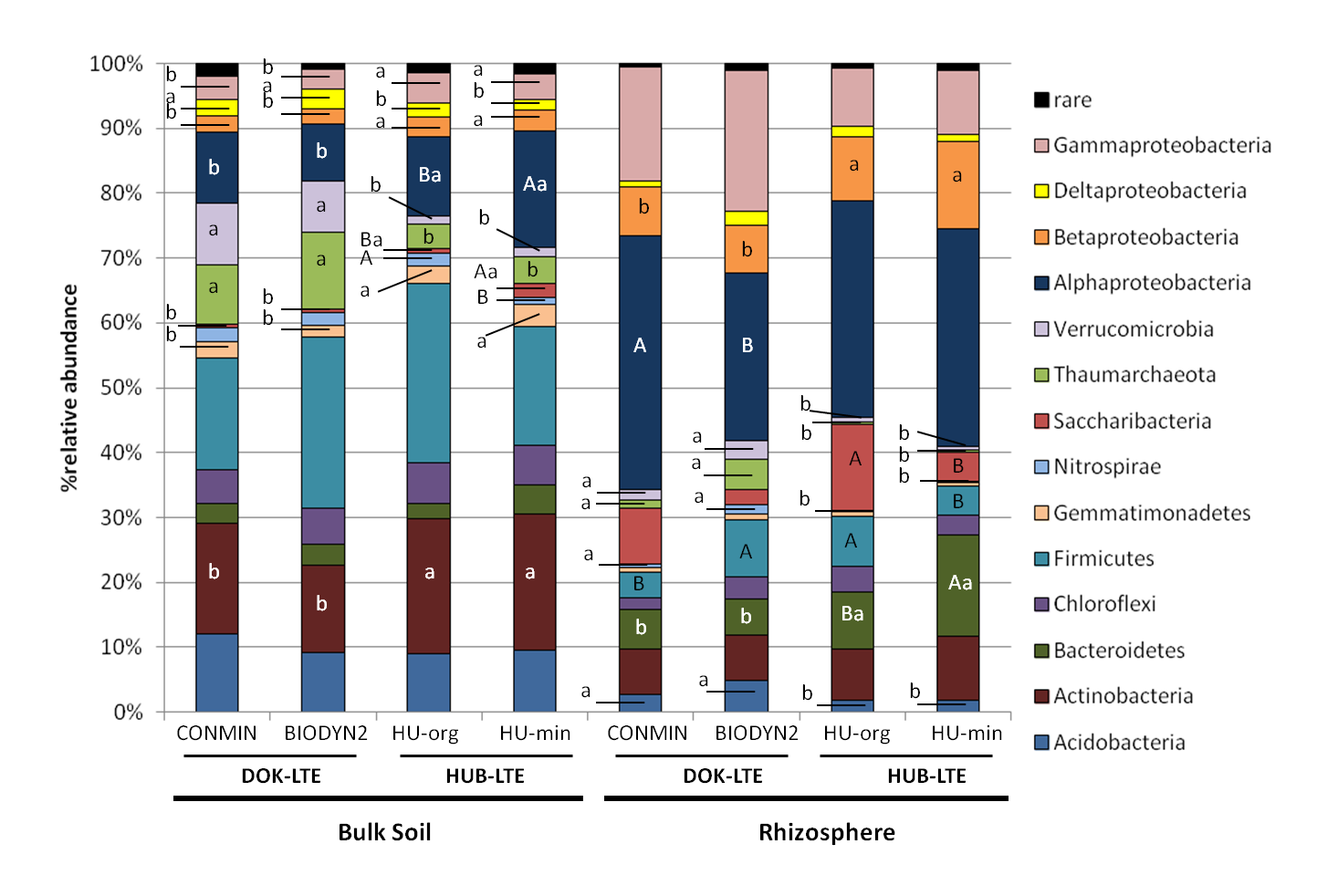


Figure S3: Prokaryotic community composition in bulk soil and rhizosphere of lettuce (rare: phyla <1% relative abundance). Different capital letters denote significant taxon differences between organic *vs.* mineral per field site and habitat. Different lowercase letters indicate significant taxon differences between field sites per habitat independent of fertilization. Missing letters indicate no significant difference (multiple pairwise, two-sided t-test, p<0.05).


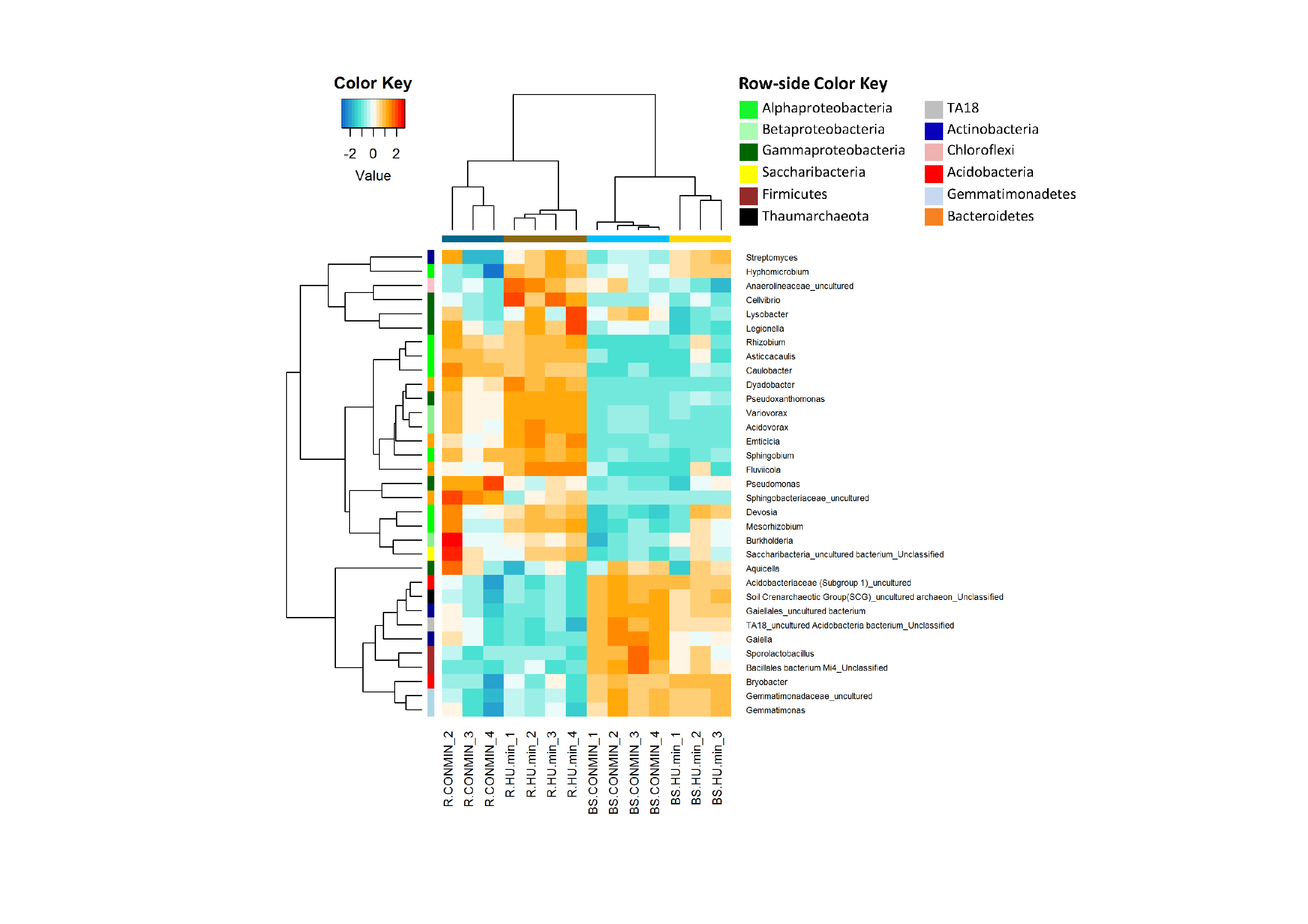
Figure S4: Heatmap showing abundance of the 20 most prevalent genera that responded significantly to the rhizosphere (Likelihood ratio test, FDR-corrected p<0.05) in both field sites under long-term mineral fertilization (CONMIN, HU-min). R- Rhizosphere, BS- Bulk Soil. Data was centered and scaled to the mean of each log-transformed taxon’s abundance. The column-side colors indicate the sample type, row-side colors indicate responder taxonomy. Seven genera belonged to significant rhizosphere responders in both field sites (see text).


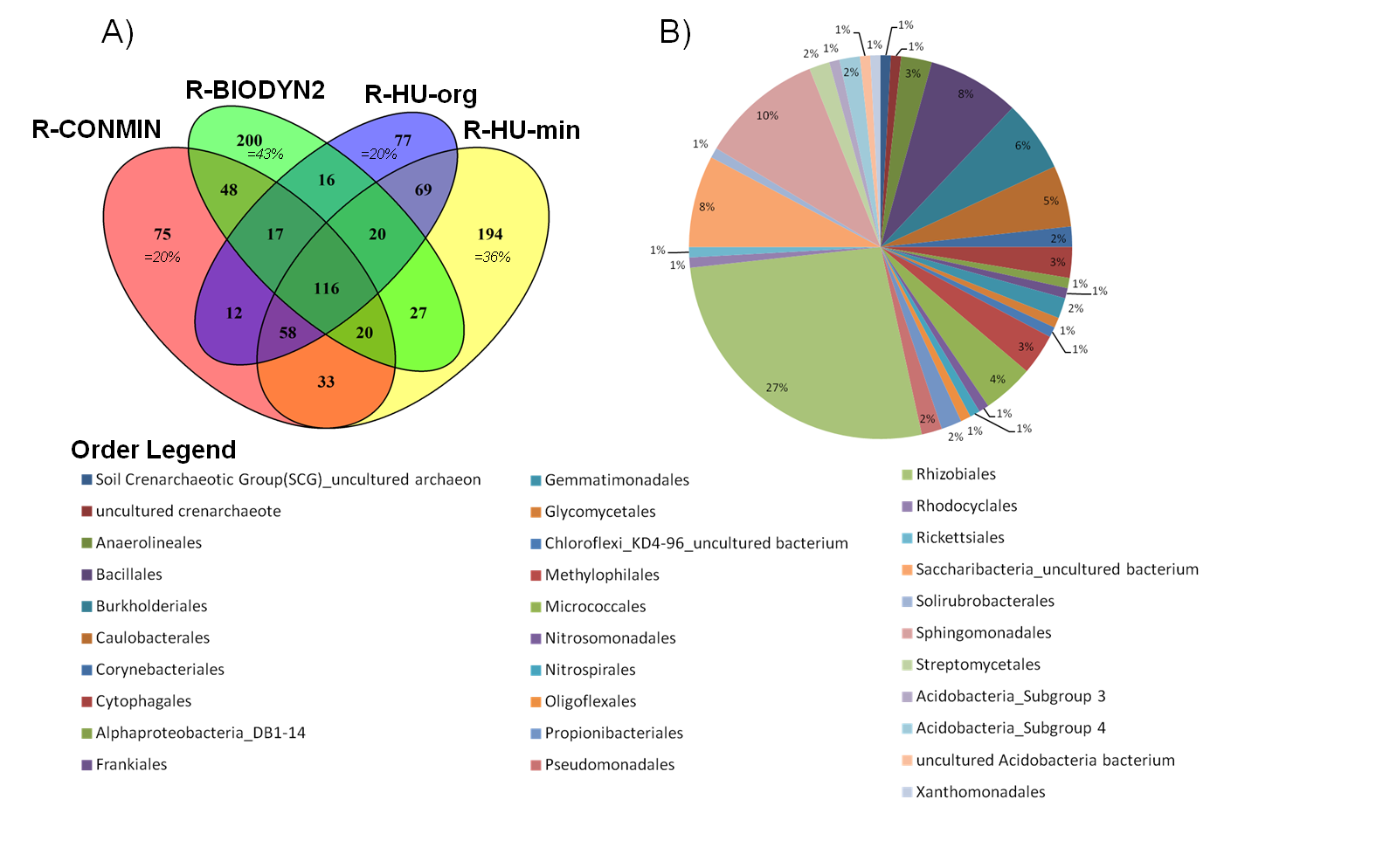


Figure S5: Lettuce rhizosphere microbiota. A) Venn Diagram showing unique and shared OTUs being present in all replicates of each soil variant. Percentages in italics indicate the proportion of treatment-dependent unique OTUs. B) Taxonomic classification on order level of 116 core OTUs present in rhizosphere samples of all soil variants.

Table S1: Overview of the used treatments as well as soil composition, annual sum of precipitation and annual mean temperature from the two long-term field experiments (LTEs) DOK-LTE and HUB-LTE.

DOK-LTE: Comparison of biodynamic, bio-organic and conventional farming systems in a 7-year crop rotation since 1978 in Therwil (CH); HUB-LTE: statische Demonstrationsanlage „Ackerbausysteme“ in Thyrow (GER).

| **Field site** | **Treatment** | **Fertilization** | **Plant protection** | **Sand**  [%] | **Silt**  [%] | **Clay**  [%] | **Precipitation**  [mm a^-1^] | **Temperature**  [°C] | **Tillage practice** | **Last field crop** |
| --- | --- | --- | --- | --- | --- | --- | --- | --- | --- | --- |
| HUB | HU-org | Organic fertilizer  (manure) | No use of pesticide | > 80^a^ | 10-14^a^ | < 5^a^ | 509.8^a^ | 9.2^a^ | Plough (25-30 cm) | Pea |
|  | HU-min | Mineral fertilizer |  |  |  |  |  |  |  | Rye |
| DOK | CONMIN | Mineral fertilizer | Use of pesticide | 15^b^ | 70^b^ | 15^b^ | 792^c^ | 9.5^c^ | Plough (20 cm) | Maize |
|  | BIODYN2 | Bio-Dynamic (manure, compost and slurry) | No use of pesticide |  |  |  |  |  |  | Maize |

^a^ Ellmer and Baumecker 2016.

^b^ Mäder *et al*., 2002

^c^ Jossi *et al*., 2009

Table S2: Analysis of prokaryotic communities from organic (BIODYN2, HU-org) or mineral (CONMIN, HU-min) fertilization from site DOK-LTE or HUB-LTE, respectively, in both habitats (bulk soil [BS] and rhizosphere [R]) based on log transformed 16S rRNA gene amplicon data (PERMANOVA analysis, 10,000 permutations, Euclidean distance).

| **Factor** | **Explained Variance [%]** | **P-Value** |
| --- | --- | --- |
| Habitat (R *vs.* BS) | 27.7 | p<0.001 *** |
| Site (DOK *vs.* HUB) | 18.5 | p<0.001 *** |
| Fertilization (Org *vs.* Min) | 7.1 | p<0.001 *** |
| Habitat x Site | 7.6 | p<0.001 *** |
| Habitat x Fertilization | 3.2 | p<0.05 * |
| Site x Fertilization | 4.5 | p<0.01 ** |
| Habitat x Site x Fertilization | 2.7 | p>0.05 |
| Residuals | 28.6 |  |

Table S3: Analysis of prokaryotic communities from organic (BIODYN2, HU-org) or mineral (CONMIN, HU-min) fertilization from site DOK-LTE or HUB-LTE, respectively, in bulk soil (BS) and rhizosphere (R) separately based on log transformed 16S rRNA gene amplicon data (PERMANOVA analysis, 10,000 permutations, Euclidean distance).

| **Factor** | **Bulk Soil** | | **Rhizosphere** | |
| --- | --- | --- | --- | --- |
|  | **Explained Variance [%]** | **P-Value** | **Explained Variance %]** | **P-Value** |
| Site (DOK *vs.* HUB) | 42.2 | p<0.001 *** | 26.2 | p<0.001 *** |
| Fertilization (Org *vs.* Min) | 15 | p<0.01 ** | 13.2 | p<0.05 * |
| Site x Fertilization | 10.1 | p<0.05 * | 9.6 | p>0.05 |
| Residuals | 32.6 |  | 51 |  |

Table S4: List of plant genes selected for expression analysis with their corresponding loci, functions in *A. thaliana* and primer sequences.

| **Name of gene (Locus tag in *Arabidopsis thaliana*)** | **Documented functions in *Arabidopsis thaliana*** | **Primer sequences (5‘ -3‘)**  All primers were designed in this study and have an annealing temperature of 55°C |
| --- | --- | --- |
| *OPT3* (AT4G16370) | Iron transporter involved in systemic iron, zinc and cadmium distribution within the plant. | OPTf - GGCTTGTCACCGGAATGATC  OPTr - TGCAAGGCGAAGAACAACAA |
| *NIA1*  (AT1G77760) | Nitrate reductase, nitrate induced expression and involved in nitrate assimilation. | NIAf - ACCTTCACCATGTCCGAAGT  NIAr - TGAGTATGCTGTCACTGCCA |
| *PR1*  (AT2G14610) | Pathogenesis related protein 1, Salicylic acid (SA) dependent expression, involved in resistance against broad spectrum of pathogen. | PR1f - GAGAAGGCCGATTATGATTA  PR1r - ATTATTGCATTGAACCCTTG |
| *PDF1.2* (AT5G44420) | Plant defensin factor involved in Jasmonic acid (JA)/ Ethylene (Et) dependent pathogen defense response. Involved in Induced systemic resistance (ISR). | PDF1.2f - ACAAGATATGCGAGCGGAGA  PDF1.2r - TGACAGGCTCCATGTTTTGC |
| *LOX1*  (AT1G55020) | Lipoxigenase; Upstream gene involved in the oxylipin metabolic pathway. Involved in the signaling of wounding response and JA induced defense against specific pathogens. | LOX1f - AAGAGCAGAAGCCACCCATA  LOX1r - GTGGAAGGAACTGCGAGAAG |
| *WRKY70* (AT3G56400) | Transcription factor involved in both SA- and JA-mediated signal pathways. Also involved in abiotic stress signaling. | WRKY70f - GCACACACAAAACCGACCAA  WRKY70r - AGTTGTTGCAAGTATGGTGTCC |
| *WRKY25* (AT2G30250) | Negative regulator of SA-mediated defense responses, elevated expression in response to oxidative stress, heat stress or wounding. | WRKY25f - TGTTCAATGAGGAAGAAGGTGG  WRKY25r - TCGTTTGGTGGATTGTGGTTT |
| *MYC2* (AT1G32640) | JA-regulated transcription regulator involved in defense against bacterial pathogens/insect herbivory, flavonoid biosynthesis, and oxidative stress tolerance. | MYCf - CGGGAGCTGAATTCATTGAT  MYCr - CTACCGTTGACGAACGACTG |
| *ERF104* (AT5G61600) | Et- Response Factor family transcription factor. Involved in Et- signaling and resistance to necrotrophic pathogens. | ERF104f - AGAGGAGTAAGACAACGGCC  ERF104r - TTTTGCTGCATCTATGGCGG |
| *CAT1* (AT1G20630) | Catalase, induced by hydrogen peroxide, abscisic acid (ABA), drought, and salt stress. | CAT1f - GGTCCAAGGCGATGTCTTTG  CAT1r - ATGAACAGCTGGCGTTTTGT |
| *PER50* (AT4G37520) | Peroxidase; Response to environmental stresses such as wounding, pathogen attack and oxidative stress. | PER50f - CTGTCAACACATGGGCTTCC  PER50r - TCCCACTTCGACCCGTTTTA |
| *ERF6* (AT4G17490) | Et- Response Factor family transcription factor. Response to oxidative stress and biotic stress induced by biotrophic and necrotrophic pathogens. | ERF6f - CAAAACGTCGCGGATCTAGG  ERF6r - GACGCAACCTCAAGTGGAAA |
| *ZAT10* (AT1G27730) | Zinc finger protein; Transcriptional repressor involved in abiotic stress responses. Positive transcriptional regulator for salinity, heat and osmotic stress. | ZATf - TCGTGACTCCTTCCACTTCC  ZATr - TAGGTGGACACAAGGCTAGC |
| *SEN1* (AT4G35770) | Senescence-associated protein DIN1; Associated with leaf senescence and decrease in photosynthesis. | SEN1f - CTGGACATGCTATTGGTGCC  SEN1r - CGAGAAATTCATCGTCTTTCCCA |
| *RbohD* (AT5G47910) | Respiratory burst oxidase homolog D. Involved in rapid reactive oxygen species (ROS) production on perception of pathogen-associated molecular patterns (PAMPs) by pattern recognition receptors (PRRs). | RbohDf - ACAGGGTTCTTTCGACTGGT  RbohDr - AATTAGAGCAGACCTGGCGT |
| *RbohF* (AT1G64060) | Respiratory burst oxidase homolog F. Involved in hypersensitive reaction (HR)-related cell death and interact with intercellular ROS regulating pathogen defense responses. | RbohFf - TCATCGGCTCTAAGAAGCCC  RbohFr - TGCTCCAGATGACGATTACCT |
| *GST6* (AT2G47730) | Glutathione S-transferase expressed in response to auxin, SA and hydrogen peroxide. JA- independent induction by 12-oxo-Phytodienoic Acid (OPDA) in plant defense. | GST6f - GCCCAAATACTTGCTCTCCG  GST6r - TTGGGATGACTACCGACGAG |
| *HSP70* (AT3G12580) | Heat shock chaperone proteins induced by high light intensity, response to hydrogen peroxide, response to heat and 12-oxo-phytodienoic acid (OPDA) induced response to wounding. | HSP70f - TTCGCCTCCACCTTCTTCTT  HSP70r - TCACCAACGACAAGGGAAGA |
| *RD22* (AT5G25610) | Dehydration responsive protein, induced by ABA in response to drought, salt and oxidative stress. | RD22f - AGTGACAGCTGCTACTCGAG  RD22r - TTCTCCGACCAAAGACACCA |
| *MYB15* (AT3G23250) | ABA inducible abiotic stress regulator, upregulated in cold and drought stress. | MYB15nf - AGGTGGGGTTGAAGAAAGGA  MYB15nr - CGTACCAGCTTTTGAAGGCA |
| *LEC* (AT3G15356) | Legume lectin –like protein, appears to play a role in the JA/Et response, chitin-elicited defense responses. | LEC1f - TCGTTCTCCACCTCTTTCGT  LEC1r - GTCCGAGATGTTGCGAAGAC |

Table S5: Primers and probes used in this study

| **Template** | **Primer/Probe** | **Primer/Probe sequence**  **(5‘ -3‘)** | **Annealing temperature [°C]** | **Reference** |
| --- | --- | --- | --- | --- |
| 16S rRNA gene (V3-V4, Illumina) | 341F | CCTAYGGGRBGCASCAG | 56 | Sundberg *et al.*, 2013 |
|  | 806R | GGACTACHVGGGTWTCTAAT |  | Caporaso *et al*., 2011 |
| bacterial 16S rRNA gene (qPCR) | Bact1369F | CGGTGAATACGTTCYCGG | 56 | Suzuki *et al*., 2000 |
|  | Prok1492R | GGWTACCTTGTTACGACTT |  |  |
|  | TM1389F (5’-FAM, 3’-TAMRA) | CTTGTACACACCGCCCGTC |  |  |
| archaeal 16S rRNA gene (qPCR) | ARC787F | ATTAGATACCCSBGTAGTCC | 60 | Yu *et al*., 2005 |
|  | ARC1059R | GCCATGCACCWCCTCT |  |  |
|  | ARC915F (5’-FAM, 3’-TAMRA) | AGGAATTGGCGGGGGAGCAC |  |  |

**References**

1. Ellmer, F., and Baumecker, M. (2016) *Versuchsführer 2016 - Lehr- und Forschungsstation Thyrow*. Humboldt – Universität zu Berlin.
2. Mäder, P., Fliessbach, A., Dubois, D., Gunst, L., Fried, P., Niggli, U. (2002) Soil fertility and biodiversity in organic farming. *Science.* **296:** 1694-1697.
3. Jossi, W., Gunst, L., Zihlmann, U., Mäder, P., and Dubois, D. (2009) DOK-Versuch: Erträge bei halber und praxisüblicher Düngung. *Agrarforschung* **16**:296-301.
4. Sundberg, C., Al-Soud, W.A., Larsson, M., Alm, E., Yekta, S.S., Svensson, B.H. *et al*. (2013) 454 pyrosequencing analyses of bacterial and archaeal richness in 21 full-scale biogas digesters. *FEMS Microbiol Ecol* **85**:612-626.
5. Caporaso, J.G., Lauber, C.L., Walters, W.A., Berg-Lyons, D., Lozupone, C.A., Turnbaugh, P.J. *et al*. (2011) Global patterns of 16S rRNA diversity at a depth of millions of sequences per sample. *Proc Natl Acad Sci USA* **108**: 4516-4522
6. Suzuki, M.T., Taylor, L.T., and DeLong, E.F. (2000) Quantitative analysis of small-subunit rRNA genes in mixed microbial populations via 5′-nuclease assays*. Appl Environ Microbiol* **66** :4605-4614.
7. Yu, Y., Lee, C., Kim, J., and Hwang, S. (2005) Group‐specific primer and probe sets to detect methanogenic communities using quantitative real‐time polymerase chain reaction. *Biotechnol Bioeng* **89**: 670-679.
